# Systematic characterization of horizontally transferred biosynthetic gene clusters in the human gut microbiota using HTBGC‐Finder

**DOI:** 10.1002/imo2.62

**Published:** 2025-02-01

**Authors:** Jiacheng Wu, Xiao Yang, Lanlan Zhao, Ziyun Li, Guoping Zhao, Lei Zhang

**Affiliations:** ^1^ Microbiome‐X, School of Public Health, Cheeloo College of Medicine Shandong University Jinan China; ^2^ CAS Key Laboratory of Computational Biology, Bio‐Med Big Data Center, Shanghai Institute of Nutrition and Health Chinese Academy of Sciences Shanghai China; ^3^ State Key Laboratory of Microbial Technology Shandong University Qingdao China

**Keywords:** biosynthetic gene clusters, gut microbiome, horizontal gene transfer, metagenomics, quorum sensing

## Abstract

The human gut microbiota contains biosynthetic gene clusters (BGCs) that encode bioactive secondary metabolites, which play pivotal roles in microbe‐microbe and host‐microbe interactions and serve as a rich source of pharmaceutical lead compounds. Understanding the horizontal transfer of BGCs can reveal insights into microbial adaptation, resource utilization, and evolutionary mechanisms, thereby advancing biotechnological applications. Despite its importance, horizontal transfer of BGCs within the gut microbiota remains poorly understood. In this study, we introduce a novel tool, the Horizontally Transferred Biosynthetic Gene Clusters Finder (HTBGC‐Finder), designed to systematically identify potential horizontally transferred BGCs (HTBGCs) within the extensive human gut microbiota. Using HTBGC‐Finder, we identified 81 potential HTBGCs, underscoring the prevalence and significance of horizontal gene transfer in shaping the genetic landscape of the gut microbiome. Remarkably, ribosomally synthesized and post‐translationally modified peptides (RiPPs) constituted the majority of these HTBGCs (76 out of 81, 93.83%), exhibiting a significantly higher transfer rate compared to non‐RiPPs (Chi‐squared test, *p* < 0.001). Upon detailed examination of BGCs, cyclic‐lactone‐autoinducer (CLA) and RiPP recognition element (RRE)‐containing BGCs were predominant, representing nearly three‐quarters of the total (45, or 55.56%, and 14, or 17.28%, respectively). Notably, CLA BGCs also demonstrated a higher transfer rate than non‐CLA BGCs (Chi‐squared test, *p* < 0.001). Taxonomy profiling revealed that horizontal BGC transfer occurred exclusively in the phyla Bacteroidota (synonym Bacteroidetes) and Bacillota (synonym Firmicutes), with 50 and 31 instances, respectively. Furthermore, cross‐phylum transfer events were observed, highlighting the complex interactions between the gut microbiota and host health. These findings offer valuable insights into the horizontal transfer dynamics of BGCs within the gut microbiome and their potential implications for host‐microbiota interactions.

## Highlights

- Introduction of HTBGC‐Finder: Developed a novel tool, the Horizontally Transferred Biosynthetic Gene Clusters Finder (HTBGC‐Finder), to identify potential horizontally transferred BGCs (HTBGCs).
- Identification of HTBGCs: Discovered 81 potential HTBGCs within the human gut microbiota using HTBGC‐Finder.
- Higher Transfer Rates: Ribosomally synthesized and post‐translationally modified peptides (RiPPs) and cyclic‐lactone‐autoinducer (CLA) BGCs exhibited significantly higher transfer rates compared to non‐RiPPs and non‐CLA BGCs.
- Phylum‐Specific Transfer: Horizontal transfer of BGCs was detected exclusively within the phyla Bacteroidota (synonym Bacteroidetes) and Bacillota (synonym Firmicutes).
- Cross‐Phylum Transfer: Observed instances of cross‐phylum horizontal transfer, suggesting a mechanism for rapid evolution within the gut microbiota.

## Graphical abstract

To investigate recently horizontally transferred biosynthetic gene clusters (HTBGCs) within individuals from metagenome‐assembled genomes (MAGs), we developed a tool named Horizontally Transferred Biosynthetic Gene Clusters Finder (HTBGC‐Finder). In an extensive analysis of human gut microbiota, HTBGC‐Finder identified 81 potential HTBGCs. Ribosomally synthesized and post‐translationally modified peptides (RiPPs) and cyclic‐lactone‐autoinducer (CLA) BGCs exhibited significantly higher transfer rates compared to non‐RiPPs and non‐CLA BGCs. Horizontal BGC transfer was detected exclusively within the phyla Bacteroidota (synonym Bacteroidetes) and Bacillota (synonym Firmicutes). Our analysis also revealed cross‐phylum transfer events, suggesting a mechanism for rapid evolution within the gut microbiota.

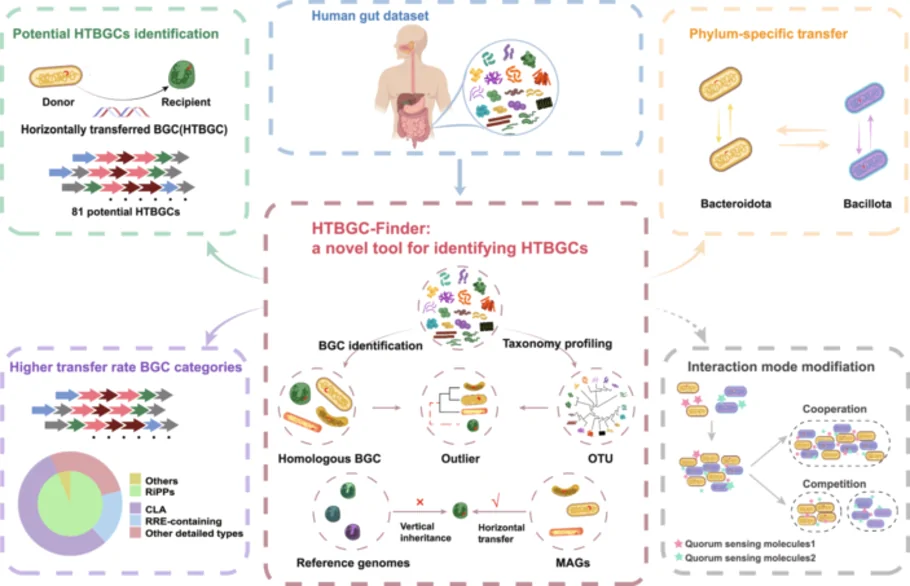

## 1 INTRODUCTION

The human gastrointestinal tract is a critical ecological niche, harboring an astounding 100 trillion microbes [1]. Collectively, these intestinal microbes possess over 3.3 million genes, approximately 100 times more than the total number of genes in the human genome [2]. This vast genetic diversity has led to the gut microbiota being referred to as the “second genome” of the human body. The dynamic interplay among the gut microbiome, the human genome, and environmental factors exerts multifaceted impacts on human health.

Embedded within the genomes of bacteria and other gut microbiota are biosynthetic gene clusters (BGCs). These essential genetic elements, organized on DNA fragments, encode the synthesis of functional substances or perform specific functions [3]. Based on the synthetic pathway and type of encoded product, BGCs can be classified into polyketide synthetase (PKS), nonribosomal peptide synthetase (NRPS), ribosomally synthesized and post‐translationally modified peptides (RiPPs), some of which containing a conserved domain named RiPP precursor recognition element [4], terpene, saccharide, and alkaloid [5]. BGCs orchestrate the production of a diverse array of secondary metabolites (SMs) during microbial growth and development processes [6]. These small molecular substances play pivotal roles in the ecology and physiology of microorganisms [7, 8], serving as a prolific source of lead compounds for various pharmaceutical applications [9], including antibiotics [10], antifungal agents [11], antitumor agents [12], and beyond.

Despite the vast potential of BGCs, experimental validation has confirmed only a small fraction (approximately 3%) of the bacterial genomic potential for SMs [13, 14]. Many microorganisms containing BGCs remain uncultivated or are unsuitable for microbial genetic manipulation [15], primarily due to factors such as nonadaptive expression, lack of conducive induction conditions, and intricate regulatory mechanisms [16]. Heterologous expression is a key strategy to overcome these challenges, although finding an ideal host often requires complex and specific conditions [17, 18]. Assessing target BGC‐host compatibility is crucial, making the investigation of horizontal gene transfer (HGT) of BGCs an important area of research [19, 20].

HGT is well‐recognized as a common phenomenon, particularly regarding the dissemination of antibiotic resistance elements [21, 22, 23]. Comparative genomic studies have also identified HGT as an important driver of BGC's evolution and adaptation [24, 25, 26]. Various computational tools have been developed to aid in HGT detection. GIST [27] and IslandViewer [28] utilize genomic sequence composition features for HGT detection, while DarkHorse [29] and HGTector [30] rely on sequence similarity (best matches). Additionally, Ranger‐DTL [31] and AnGST [32] employ explicit phylogenetic methods to predict HGT by aligning gene trees with respective species trees.

Despite advances in HGT detection, the horizontal transfer of BGCs remains an emerging field. The evolution of BGCs in the metagenome‐assembled genomes (MAGs) has not been fully characterized. Understanding the horizontal transfer of BGCs is crucial for comprehending their spread and evolution in natural settings, as well as for determining the extent of gene exchange among strains possessing specific biosynthetic capabilities. This knowledge facilitates the selection of optimal strains for heterologous expressing desired products and valuable compounds.

In this study, we present the Horizontally Transferred Biosynthetic Gene Clusters Finder (HTBGC‐Finder), a computational tool specifically designed to identify recently horizontally transferred BGCs (HTBGCs) directly from MAGs within individuals. The HTBGC‐Finder is an automated tool that detect potential HTBGCs from MAGs with minimal human intervention, and is easy to install and operate. Using HTBGC‐Finder, we systematically analyzed potential horizontal transfer events of BGCs in the human gut, providing insights into their distribution and evolution at the intra‐individual level. Our findings enhance the understanding of microbial community adaptation, resource utilization, and BGC propagation, offering valuable insights for the development of new biotechnological applications.

## 2 RESULTS

### Development of HTBGC‐Finder

**Figure 1:**
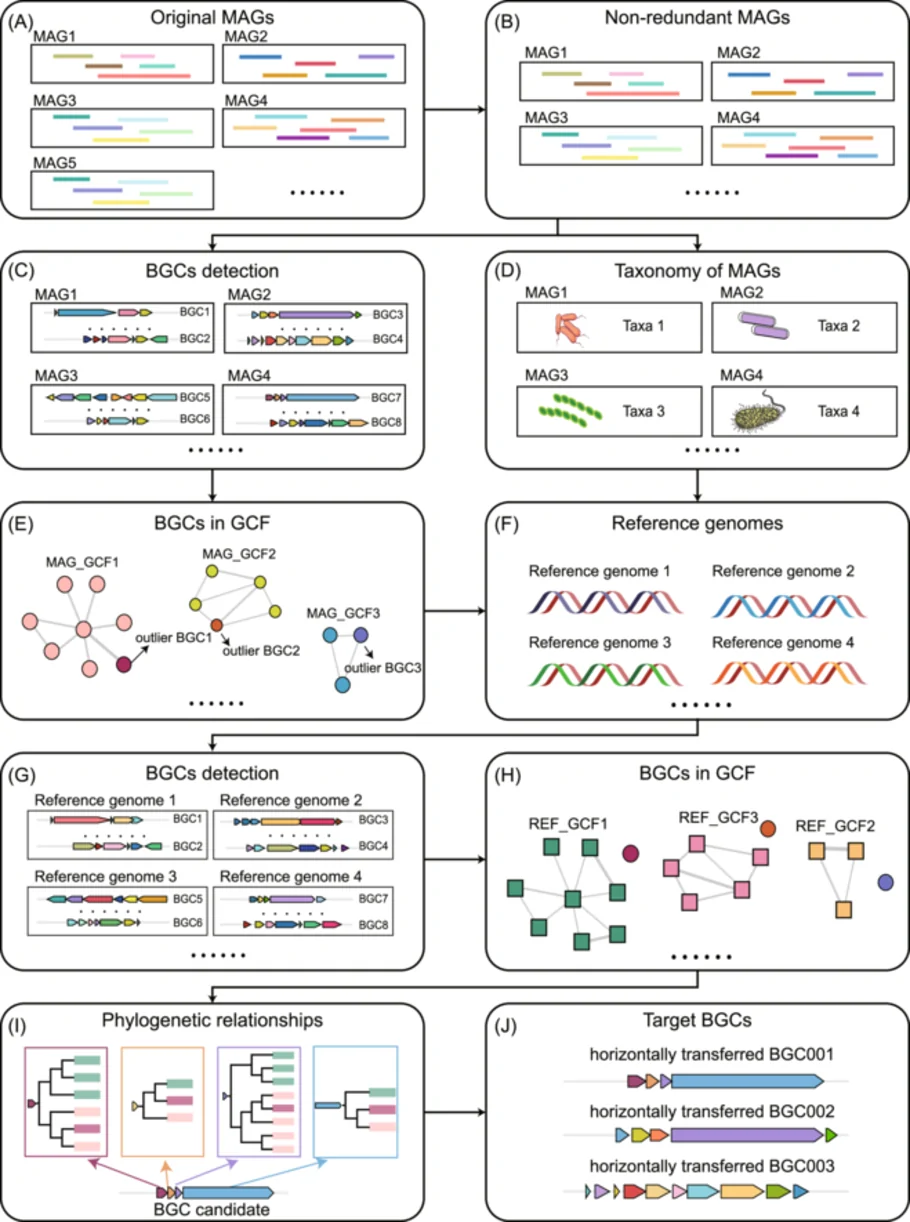
Overview of the HTBGC‐Finder. (A) The input file to HTBGC‐Finder consists of metagenome‐assembled genomes (MAGs). (B) Next step is the removal of redundant MAGs based on average nucleotide identity (ANI). (C) Then, the biosynthetic gene clusters (BGCs) contained in MAGs are identified. (D) Taxonomy profiling of different MAGs is analyzed. (E) Next step is construction of gene cluster family (GCF) network based on BGC similarity, and the outlier BGC within each GCF is identified according to taxonomy profiling. (F) Next step is the retrieval of outlier BGC's corresponding reference genomes. (G) Then, the BGCs contained in reference genomes are identified. (H) Outlier BGC and BGCs identified from reference genomes are clustered into GCF, and BGCs remain isolated are potential horizontally transferred. (I) Phylogenetic relationships of each gene in BGC candidates are performed and statistically analyzed. (J) Outlier BGCs with phylogenetic distance from MAGs closer than reference genomes are inferred to be horizontally transferred.

HTBGC‐Finder is a computational tool designed to identify recently horizontally transferred BGCs within individuals from MAGs. The tool's schematic overview is depicted in Figure 1. Key components of HTBGC‐Finder include: (1) BGCs Identification and Taxonomy Profiling: Nonredundant MAGs are processed to identify BGCs, followed by taxonomy profiling to remove redundant genomes and ensure accurate analysis. (2) Construction of Gene Cluster Family (GCF) Network: Identified BGCs are clustered into different GCFs based on similarity, facilitating the study of BGC distribution and evolution across different taxonomy groups. (3) Identification of Outlier BGCs: Outlier BGCs, which differ in taxonomy from others within the same GCF, are preliminarily flagged as potential horizontally transferred BGCs. (4) BGCs Identification from Reference Genomes: Outlier BGCs are traced back to their respective MAGs, and reference genomes with matching taxonomy profiles are retrieved from the National Center for Biotechnology Information (NCBI). BGCs from these reference genomes are then identified. (5) Construction of GCFs from MAGs and Reference Genomes: MAGs with BGCs matching the same GCF as outliers and reference genomes to the same taxonomy of outliers are grouped. Potential HTBGCs are inferred from the isolation of outlier BGCs. (6) Phylogenetic Analysis: Maximum likelihood (ML) phylogenetic reconstruction is performed to compare the phylogenetic relationships between MAGs and reference genomes to infer horizontal transfer.

The HTBGC‐Finder takes MAGs as input and produces a tabular output with information on BGC origin, phylogenetic distance, statistical *p* values, HTBGC potential, BGC length, type, recipient, potential donor, and similar BGCs within the same genus. HTBGC‐Finder is available for application to metagenomic datasets (https://github.com/Shirly-Yang/HTBGC-Finder, see “Data availability statement” for repository and installation details).

### Validation of HTBGC‐Finder

**Figure 2:**
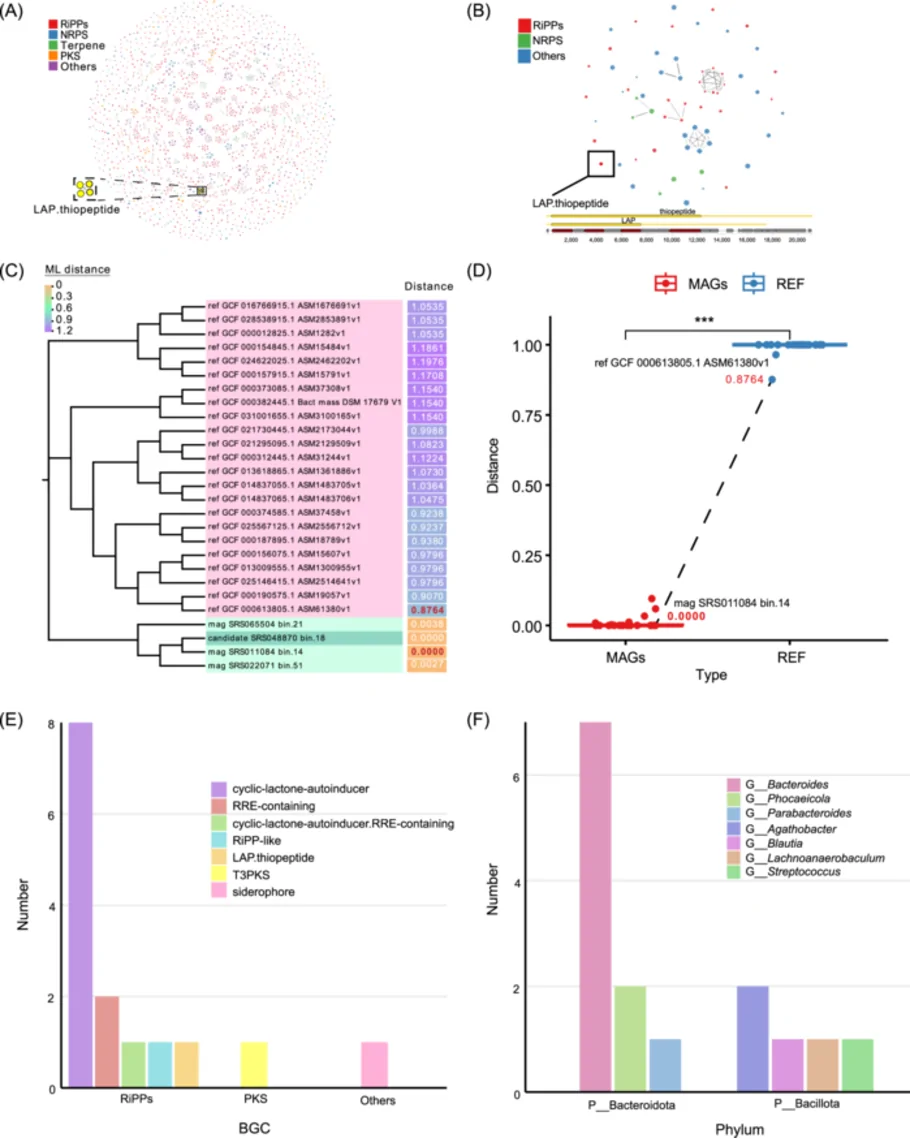
Detection of potential horizontally transferred thiopeptide. (A) The gene cluster family (GCF) network constructed by biosynthetic gene clusters (BGCs) identified from 1150 metagenome‐assembled genomes (MAGs). The GCF containing target thiopeptide was highlighted in yellow. (B) The GCF network derived from clustering the target thiopeptide and BGCs within the reference genomes of corresponding taxonomy. The target thiopeptide contains 12 genes. (C) Maximum likelihood (ML) phylogeny of the target thiopeptide. ML phylogenetic reconstruction was performed using twelfth gene of thiopeptide from SRS048870_bin18_contig135 as the query gene, with the reference genomes of the corresponding taxonomy and MAGs within the same GCF as query clusters. The reference genomes are represented by the RefSeq genome accession number and the corresponding Assembly number. Metagenomic data is represented by MAG accession number. (D) Comparison of the phylogenetic distance from MAGs and reference genomes. Each dot represents a gene of the target thiopeptide. “MAGs” group represents BGCs from MAGs, “REF” group represents BGCs from reference genomes, and “Distance” represents phylogenetic distance from the target thiopeptide. The statistical difference between the two groups was compared through the paired Wilcoxon test. ***p < 0.001. (E) Type of identified potential horizontally transferred BGCs. (F) Taxonomic profiling of identified potential horizontally transferred BGCs. NRPS, nonribosomal peptide synthetase; RiPP, ribosomally synthesized and post‐translationally modified peptide; PKS, polyketide synthetase; RRE, RiPP recognition element.

To evaluate the recognition performance of HTBGC‐Finder, we applied it to detect the potential HTBGCs involved in thiopeptide production in metagenomic samples from the Human Microbiome Project (HMP), as detailed in previous publications [33], allowing for the benchmarking of our approach. We retrieved the necessary raw data associated with the thiopeptide from the article (Table S1), followed by assembly and binning processes, resulting in 1150 bins based on the BioSample. Subsequently, we analyzed the data set using HTBGC‐Finder with the default parameters, which led to the identification of potential HTBGCs, including the reported thiopeptide from SRS048870_bin18 (Figure 2, Table S2). Among the 1150 bins, 1840 BGCs were clustered into 1035 GCFs based on similarity. Notably, the thiopeptide BGC from SRS048870_bin18 clustered together with BGCs from SRS022071_bin51, SRS011084_bin14, and SRS065504_bin21 into the same GCF (Figure 2A). These bins were classified into different taxonomic groups, with SRS048870_bin18 classified as Bacteroidota, while the others classified as Bacillota. The distinct phylogenetic distance of SRS048870_bin18 suggests its BGC is a candidate for horizontal transfer.

HTBGC‐Finder traced the taxonomy of SRS048870_bin18 to 23 reference genomes from NCBI (Table S3). Clustering of the thiopeptide and reference genome BGCs into GCFs revealed the thiopeptide remained isolated, not clustering with other BGCs into the same GCF (Figure 2B), indicating potential horizontal transfer.

To investigate this possibility, HTBGC‐Finder employed phylogenetic analysis, revealing a closer phylogenetic proximity of the thiopeptide and MAGs group to reference genomes group (Figure 2C), with the difference being statistically significant (*p* < 0.001) (Figure 2D). This result suggests a closer association of this thiopeptide BGC with MAGs and a higher likelihood of horizontal transfer rather than vertical inheritance.

Additionally, we conducted an average nucleotide identity (ANI) analysis from both the reference genomes and MAGs clustered with the thiopeptide into the same GCF (Table S4). The analysis revealed that SRS048870_bin18, to which the thiopeptide belongs, had the highest ANI (93.019%) with *Phocaeicola dorei* DSM 17855^T^ (RefSeq assembly accession: GCF_000156075.1), implying that *Phocaeicola dorei* is the closest species to SRS048870_bin18 (Figure S1).

Following a similar approach, HTBGC‐Finder identified a total of 15 potential HTBGCs from the data set, including 13 RiPPs, 1 PKS, and 1 classified into “Others.” Among RiPPs, in addition to the identified thiopeptide, 8 cyclic‐lactone‐autoinducer (CLA), 2 RiPP recognition element (RRE)‐containing, 1 hybrid containing CLA and RRE‐containing, and 1 RiPP‐like were identified (Figure 2E, Table S2). Taxonomically, 10 BGCs were attributed to Bacteroidota (synonym Bacteroidetes) and 5 to Bacillota (synonym Firmicutes) (Figure 2F, Table S2).

### Evaluation of HTBGC‐Finder's performance

**Figure 3:**
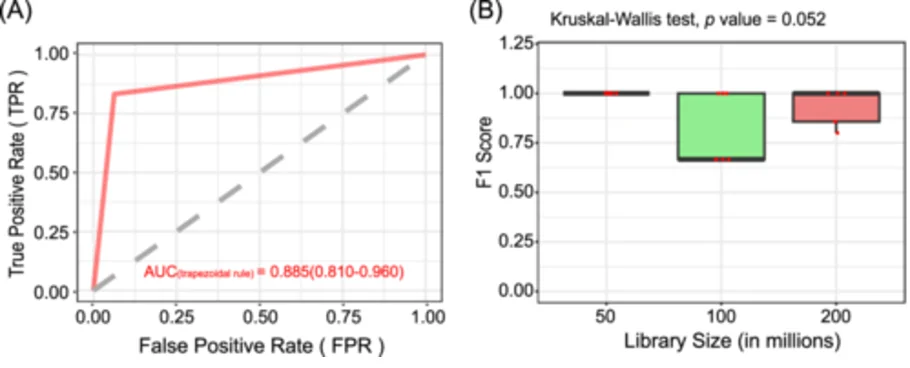
Predictive performance of HTBGC‐Finder. (A) Receiver operating characteristic (ROC) curve illustrating the prediction performance of HTBGC‐Finder on simulated metagenomes, with the area under the curve (AUC) (trapezoidal rule) 0.885 (95% confidence interval [CI]: 0.810–0.960 (DeLong)). (B) Box plot is used to show distributions of F1 scores across various simulated metagenome library sizes. No significant correlation was found between F1 scores and library sizes 50 M, 100 M, and 200 M (Kruskal–Wallis test, *p* = 0.052).

To further evaluate the performance of HTBGC‐Finder, we simulated BGC transfers across different taxonomic levels and observed whether the tool detects the intended transfer events. Specifically, we selected 20 reference genomes of keystone species in the human gut microbiome [34, 35] and evaluated the HTBGC‐Finder prediction performance on simulated metagenomes of which the HTBGCs are known. The predicted HTBGCs were then compared with the actual HTBGCs to obtain prediction accuracy. The receiver operating characteristic (ROC) curve demonstrated that HTBGC‐Finder achieved high sensitivity and specificity, with an area under the curve (AUC) of 0.885 (95% confidence interval [CI]: 0.810–0.960 (DeLong)). This suggests that HTBGC‐Finder is highly effective in distinguishing between true and false positives in predicting HTBGCs across different metagenomic datasets (Figure 3A, Table S5).

We next investigated whether HTBGC‐Finder accuracy is influenced by inherent characteristics of the simulated metagenomes, specifically focusing on library size. Our analysis revealed that the F1 scores for BGC prediction were generally independent of library size 50 million, 100 million, and 200 million (Kruskal–Wallis test, *p* = 0.052) (Figure 3B, Table S6). This suggests that HTBGC‐Finder's performance remains consistent across varying library sizes, indicating its robustness in handling metagenomes with different sequencing depths. These results underscore the robustness of HTBGC‐Finder in accurately predicting HTBGCs, making it a valuable tool for the detection of HGT in gut microbiomes.

### Systematic horizontal transfer of BGCs in human gut microbiota

To delve into the intricate phenomenon of horizontal transfer of BGCs within the human gut microbiota, we employed HTBGC‐Finder to analyze an extensive data set from human gut microbiota [2], which reconstructed 92,143 MAGs from 11,850 human gut microbiomes. To comprehensively analyze the horizontal transfer of BGCs within each sample, we retrieved and classified these MAGs according to the BioSample accession number, resulting in 5918 qualified samples with varying numbers of MAGs (Table S7). Subsequently, we selected samples corresponding to 20 or more MAGs to systematically characterize the potential HTBGCs among them.

**Figure 4:**
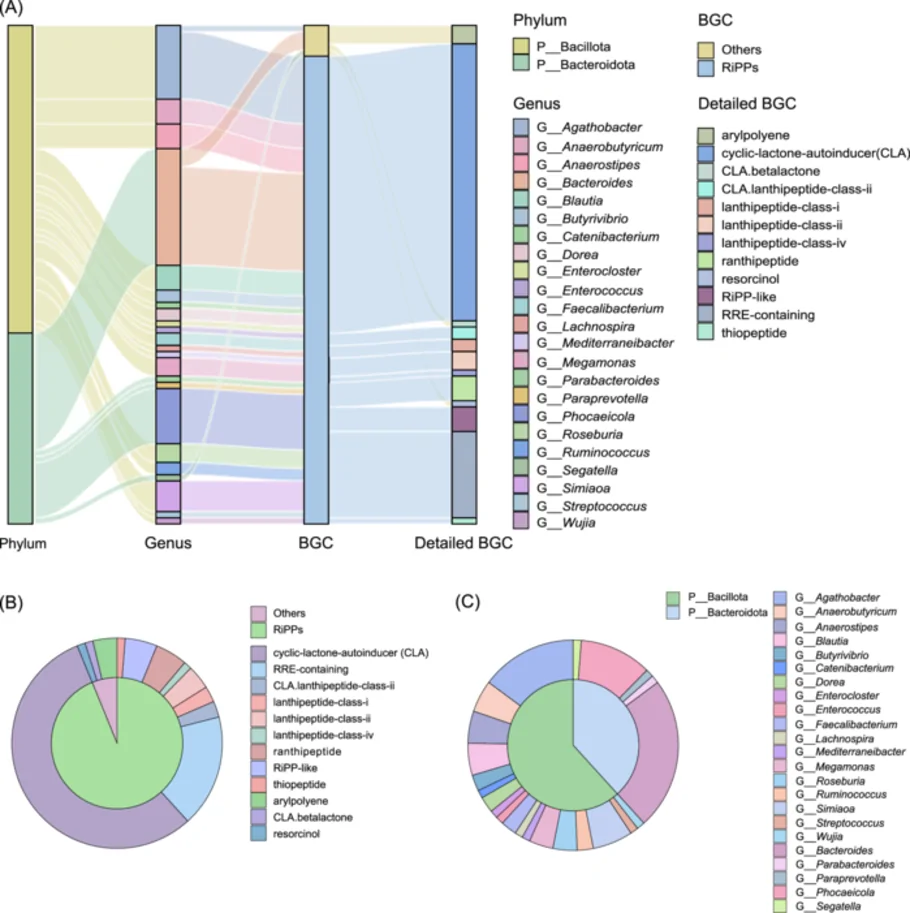
Potential horizontally transferred biosynthetic gene clusters (HTBGCs) identified by HTBGC‐Finder from human gut metagenome. (A) Composition and correlation of taxonomy profiling and types of potential HTBGCs. (B) Type of potential HTBGCs. (C) Taxonomy profiling of potential HTBGCs. RiPP, ribosomally synthesized and post‐translationally modified peptide; RRE, RiPP recognition element.

As a result, HTBGC‐Finder identified 81 BGCs that potentially underwent horizontal transfer in the human gut microbiota (Table S8). Our analysis of the taxa to which these BGCs belong revealed that Bacteroidota (synonym Bacteroidetes) and Bacillota (synonym Firmicutes) were the only two phyla where BGC horizontal transfer occurs, with 50 and 31, respectively. At the genus level, *Bacteroides* (19 of 81) and *Agathobacter* (12 of 81) exhibit the highest occurrence of BGC horizontal transfer (Figure 4, Table S9).

To further explore the shared characteristics of potential HTBGCs, we investigated their types and functions. Among the 81 identified BGCs, RiPPs constituted the predominant type (76 of 81), accounting for 93.83% of the identified BGCs, followed by arylpolyene (3 of 81) at 3.70%. Additionally, one resorcinol and one hybrid BGC containing CLA and betalactone each represented 1.23% (Figure 4). More specifically, CLA and RRE‐containing accounted for nearly three‐quarters of the total number, with 45 (55.56%) and 14 (17.28%) BGCs, respectively (Figure 4).

**Figure 5:**
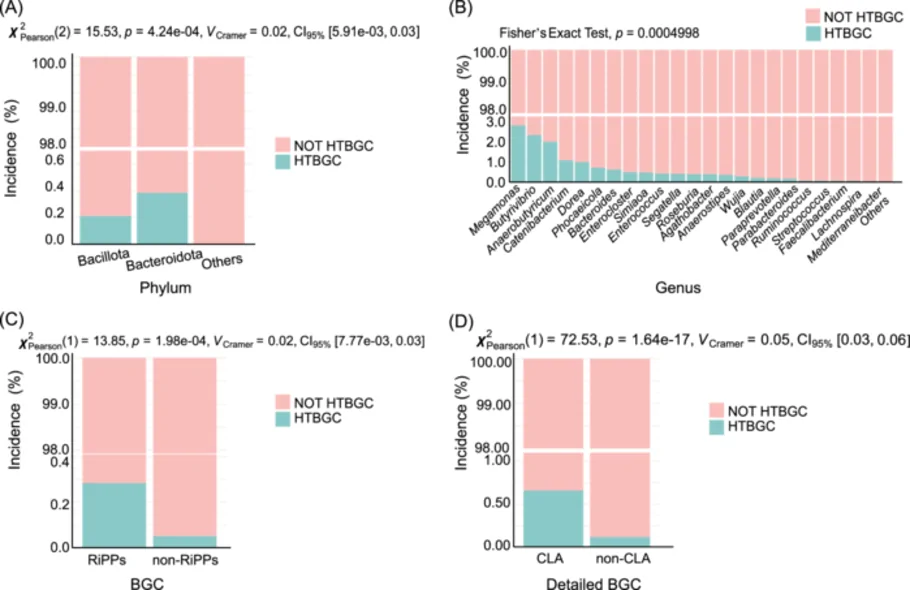
Incidence of potential horizontally transferred biosynthetic gene clusters (HTBGCs) identified from human gut microbiome. (A) Comparison of potential HTBGCs among different phyla. (B) Comparison of potential HTBGCs among different genera. (C) Comparison of potential HTBGCs between ribosomally synthesized and post‐translationally modified peptide (RiPP) and non‐RiPP. (D) Comparison of potential HTBGCs between cyclic‐lactone‐autoinducer (CLA) BGCs and non‐CLA BGCs. Group “NOT HTBGC” represents BGCs that may not be acquired through horizontal transfer. Group “HTBGC” represents BGCs inferred to be horizontally transferred.

To gain a comprehensive understanding of the horizontal transfer phenomena of BGCs, we conducted an analysis of transfer rates. The results showed that Bacteroidota (30 of 7732) exhibited a horizontal transfer rate of 0.39%, followed by Bacillota (49 of 22,731) at 0.22% (Figure 5A, Table S9), with statistically significant differences between different phyla (Pearson's Chi‐squared test, *p* < 0.001). Regarding genera, those that did not detect HTBGC were categorized as “Others.” Notably, *Megamonas* exhibited the highest transfer rate at 2.68% (3 of 112), followed by *Butyrivibrio* at 2.22% (2 of 90), *Anaerobutyricum* at 1.91% (4 of 209), and *Catenibacterium* at 1.04% (1 of 96). Fisher's exact test revealed significant differences among groups (*p* < 0.001), as shown in Figure 5B. Further analysis showed that the horizontal transfer rate of RiPPs was the highest (77 of 27,152), with a significant difference observed compared to non‐RiPPs groups (Pearson's Chi‐squared test, *p* < 0.001) (Figure 5C, Table S9). Additionally, the horizontal transfer rate of CLA and CLA‐related (referring to hybrid BGCs containing CLA) was the highest (48 of 7267, 2.94%), with statistical differences observed compared to non‐CLA related groups (Pearson's Chi‐squared test, *p* < 0.001) (Figure 5D, Table S9).

**Figure 6:**
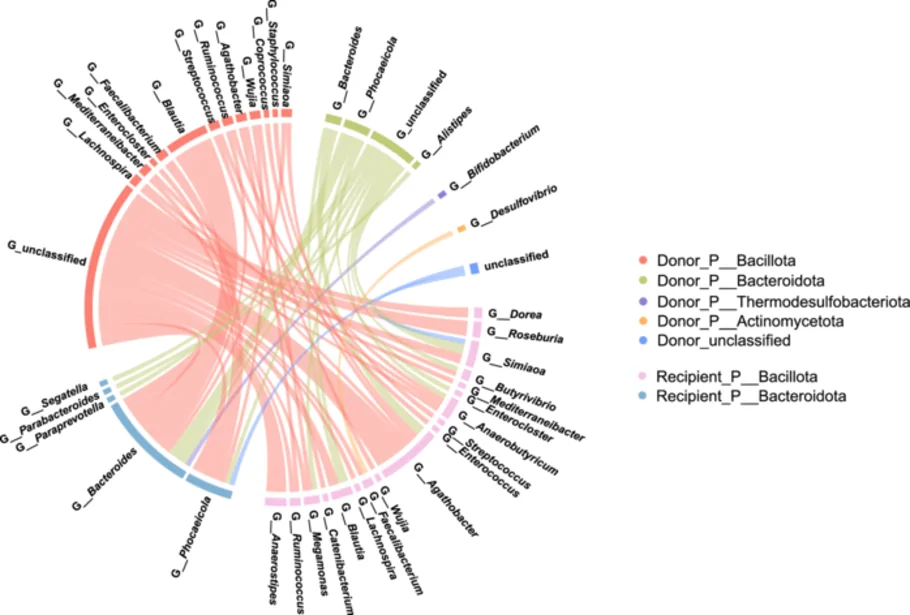
Recipients and potential donors of potential horizontally transferred biosynthetic gene clusters (HTBGCs). The chord plot showed the taxonomy profiling of recipients and potential donors of HTBGCs. Those not classified into any category were grouped as “unclassified.”

To further investigate potential patterns of BGC horizontal transfer and understand the trends and direction of transfer, we conducted an analysis targeting recipients and potential donors of HTBGCs. In addition to transfers occurring within the same phylum, we observed instances of potential horizontal transfer between Bacillota and Bacteroidota, as well as transfers from Bifidobacterium and Desulfovibrio to Bacteroidota (Figure 6).

## 3 DISCUSSION

Understanding the natural horizontal transfer of BGCs in microbiota is crucial for elucidating microbial community dynamics, BGCs evolution, and their biotechnological potential, such as identifying suitable host microorganisms for heterologous expression. HGT among closely associated bacteria in metagenomic samples can significantly impact the evolution and spread of BGCs, and bioinformatics tools are crucial for studying these processes. We introduced HTBGC‐Finder, a computational tool designed to characterize horizontal transfer events of BGCs in metagenomic datasets. When evaluating the performance of HTBGC‐Finder, the simulation process of generating reads and MAGs may introduce inherent biases, including incomplete genome recovery during assembly, fragmentation of BGCs in the reassembled MAGs, and uneven coverage caused by read simulation and binning. These factors may result in the failure prediction of certain BGCs by antiSMASH, particularly those located in low‐abundance or highly fragmented genomic regions, resulting in valid F1 scores only for a subset of genomes. Subsequent analyses were therefore limited to these genomes. Despite these challenges, the investigation into the influence of library size revealed that HTBGC‐Finder's F1 score was not significantly affected by sequencing depths, demonstrating its robustness. Additionally, the high AUC from the ROC curve highlights HTBGC‐Finder's potential as a reliable tool for HTBGC detection.

HTBGC‐Finder's ability to identify HTBGCs provides valuable insights into gut microbiomes. By applying HTBGC‐Finder to human gut metagenomes, we identified 81 potential HTBGCs, confirming the natural occurrence of BGC horizontal transfer and its role in expanding BGC diversity. Our results show that RiPPs exhibit distinct horizontal transfer patterns compared to other BGCs. RiPPs are modular macromolecular complexes that facilitate the assembly of acyl substrates into diverse bioactive peptides [36], playing crucial roles in microbial biology and pharmaceutical applications [37, 38]. Several factors may contribute to the prevalence of RiPPs in horizontal transfer: (1) Size and Complexity: RiPPs are generally smaller and less complex than other BGCs, making them more amenable to horizontal transfer via mobile genetic elements. (2) Modular Structure: The modular nature of RiPPs allows for the exchange of specific gene cluster segments, enhancing their potential for horizontal transfer. (3) Genetic Features: RiPPs may possess genetic elements like regulatory sequences and mobile elements that facilitate their horizontal movement. (4) Functional Advantage: RiPPs encode bioactive compounds that are essential for bacterial survival and environmental adaptation, providing a competitive edge that promotes their spread.

The transfer of RiPPs provides rich research material for synthetic biology. The combination of RiPPs transfer and synthetic biology may advance microbial therapies and drug discovery in several ways [39, 40]. Understanding the mechanisms of RiPPs transfer can also offer new perspectives for ecological and evolutionary biology research, helping to reveal how microbial communities interact and adapt through RiPPs, thus furthering the study of their role in natural selection [41]. Additionally, by engineering microbes to release specific RiPPs, researchers can regulate the composition of microbial communities, promoting the growth of beneficial microbes while inhibiting the proliferation of pathogenic microbes [42]. This RiPPs‐based therapy may could be used to treat diseases related to microbial dysbiosis, providing more precise treatment options. More importantly, the diversity of RiPPs make them an important source for new drug development [43]. By combining methods from synthetic biology, researchers can synthesize new RiPPs or their derivatives to explore their biological activities [44], which not only aids in the discovery of new antibacterial agents but may also lead to the development of other classes of therapeutic drugs, such as antiviral, anticancer, or immunomodulatory agents [45].

Among the BGC types, CLA BGCs exhibited the highest incidence of potential horizontal transfer among detailed BGC types. Linked to quorum sensing [46, 47], a mechanism by which bacteria exchange information, coordinate behavior, and regulate gene expression [48], CLA encodes molecules that act as autoinducers, signaling bacterial presence and density [49]. The frequent horizontal transfer of CLA BGCs suggests their crucial role in fostering microbial cooperation and coordination. By regulating group behavior and modulating quorum sensing signals, CLA BGCs contribute to microbial fitness, adaptability, and ecological balance, enhancing the resilience and functional diversity of microbial communities.

Our analysis revealed that Bacillota and Bacteroidota, which together constitute nearly 50% of the human gut microbiota, have the highest incidence of horizontally transferred BGCs. These phyla are vital for intestinal health, carbohydrate fermentation, and short‐chain fatty acids production [50, 51, 52]. The acquisition of quorum sensing‐associated BGCs may provide these bacteria with enhanced communication and metabolic regulation capabilities, contributing to microbiota stability and host health. These bacteria with high propensity for horizontal transfer may possess some genomic features like repetitive sequences and specialized gene transfer systems that facilitate this process.

While HTBGC‐Finder efficiently identifies potential HTBGCs in metagenomic data through comparative genomic and phylogenomic analyses, several limitations must be acknowledged. Incorrectly assembled MAGs may contain genes not present in the original microbiota, potentially leading to erroneous conclusions about BGC transfers [53]. Additionally, this tool relies on well‐annotated genomic reference libraries, which may limit its effectiveness when analyzing samples with poorly annotated bacteria. HTBGC‐Finder also does not account for incomplete transfers or post‐transfer recombination of BGCs, which may affect their functional integrity. Future studies targeting core biosynthetic genes within BGCs could help address these issues. Despite these limitations, HTBGC‐Finder represents a significant advancement in the large‐scale identification of potential HTBGCs. However, gaining mechanistic insights into horizontal transfer processes remains complex and requires further research to refine methodologies and deepen our understanding of BGC dynamics in microbial ecosystems.

## 4 CONCLUSION

The HTBGC‐Finder introduced in this study automates the high‐throughput identification of potential HTBGCs within MAGs, providing a user‐friendly and analytically flexible tool for metagenomic research. This tool offers a robust methodology for detecting horizontally transferred BGCs, making it a valuable asset for studying microbial gene exchange. Our comprehensive analysis using HTBGC‐Finder identified 81 potential HTBGCs in the human gut microbiota. Among them, RiPPs, particularly CLAs associated with quorum sensing, exhibit the highest transfer frequency. Furthermore, Bacillota and Bacteroidota were identified as the phyla with the most frequent horizontal transfer events, including cross‐phylum exchanges. These findings shed light on the genetic exchange dynamics within microbial communities, contributing to a deeper understanding of gut microbiota and its impact on host health. Furthermore, the results have implications for synthetic biology, biosynthetic strategy optimization, and drug development. By elucidating horizontal transfer mechanisms and their effects on microbial ecosystem functionality, future research can further refine our understanding of these processes and enhance biotechnological applications.

## 5 METHODS

### Acquisition of public data

We retrieved the metagenomic sequencing data and collected the corresponding metadata based on the publication by Donia et al. [33], and detailed BioSample accession numbers could be found in Table S1. For human gut data set, we retrieved data set from human gut microbiota based on the publication by Almeida et al. [2].

### Analysis of nonredundant MAGs

We used the metaWRAP pipeline (V1.2.1) [54] to construct MAGs from combined decontaminated sequencing reads across all samples. Input sequencing data are initially assessed for sequencing‐reads‐quality grouped by module “read_qc.” Assemblies were performed using module “metaspades,” and distinct bins were identified based on module “metabat2.” After that, we used fastANI (V1.33) [55] with a threshold of 99% to remove redundancy from these MAGs. Then, we used antiSMASH (V6.1.1) [56] to identify BGCs within the obtained nonredundant MAGs. Next, we used kraken2 (V2.1.2) [57] with default parameters to perform the taxonomic identity of each MAG. Finally, to analyze homologous BGCs in nonredundant MAGs, we used BiG‐SCAPE (V1.1.5) [58] with cutoffs 0.3 to cluster BGCs into GCFs for subsequent analysis.

### Identification of the outlier BGCs within the GCF

Based on the GCF network, we identified outlier BGCs using a genetic distance matrix between different BGC genomes. Specifically, we quantified genetic distance based on the minimum identical taxa shared by BGC genomes.

For two genomes *BGC*_*i*_ and *BGC*_*j*_, genetic distance is calculated based on their match across different taxonomic levels, including Domain (D), Phylum (P), Class (C), Order (O), Family (F), Genus (G), and Species (S), represented as taxo = {D, P, C, O, F, G, S}. Starting from the most specific level (Species) to the least specific (Domain), we checked each taxonomic level for matches between genomes.

Let index(*t*) denote the position of a taxonomic level *t* in the sequence (e.g., Species has index 6 as it is the most specific level). If genomes *BGC*_*i*_ and *BGC*_*j*_ match at taxonomic level *t*, their genetic distance is computed as:

$$D_{\mathrm{ij}}=\left\{\begin{array}{l} 2^{\left(7-\mathrm{index}\left(t\right)\right)},\;\text{if}\;\exists \;t\;\in \;\text{taxo},\;\text{such that}\;{\text{classification}}_{i}\left(t\right)={\;\text{classification}}_{j}(t)\\ 999,\text{if}\;\forall \;t\;\in \text{taxo},\;{\text{classification}}_{i}\left(t\right)\;\neq \;{\text{classification}}_{j}(t) \end{array}\right.,$$

where ${\text{classification}}_{i}\left(t\right)\;$represents the taxonomic classification of genome *BGC*_*i*_ at level *t*.

For each genome *BGCi*, its average genetic distance to all other genomes was calculated as:

$${\overset{®}{D}}_{i}=\frac{1}{n-1}\;\sum_{j\neq \;i}D_{\mathrm{ij}}.$$

The overall average genetic distance across all genomes was calculated as:

$${\bar{D}}_{\mathrm{all}}=\frac{1}{n(n-1)}\;\sum_{i=1}^{n}\sum_{j>i}D_{\mathrm{ij}}.$$

For outlier BGC identification, a genome *BGCi* is considered an outlier if:

$$D_{i}>2\;{\bar{D}}_{\mathrm{all}}.$$

Alternatively, outliers can be detected based on the Z‐score:

$$Z_{i}=\frac{{\overset{®}{D}}_{i}-\mu }{\sigma },$$

where μ is the mean of all average genetic distances, and σ is the standard deviation, and *BGCi* is considered an outlier if $Z_{i}$ > 2.

### Comparison of the BGCs from MAGs and reference genomes

We used ncbi‐genome‐download (V0.3.1) to retrieve reference genomes for the genus of the BGC's source strain. This included genomes from the strain of its species and all type strains classified within the genus. Due to varying numbers of genomes in different taxonomic units, the quantity and types of reference genomes downloaded for outlier strains are not identical. For genus‐level comparison, we downloaded genomes of the same species and type strains within the genus. When there are few type strains, researchers can adjust which strains to download by modifying the command line parameters. Then we identified all BGCs in the reference genomes by antiSMASH (V6.1.1) [56]. Subsequently, the similarity between the two was compared using BiG‐SCAPE (V0.1.5) [58] with the same parameter as before.

### Phylogenetic analysis

The evolutionary relationships for target BGCs were evaluated based on DNA sequence alignments of each gene constructed with corresponding reference genomes and bins. Specifically, for each gene *g* of the target BGC, maximum likelihood (ML) phylogeny was performed using FastTree (V2.0.0) [59] to calculate the phylogenetic distance matrix between homologous BGCs identified in both reference genomes and bins:

$$D_{\mathrm{phylogenetics}}^{\mathrm{ref\_genomes},\;\mathrm{bins}}(g).$$

The minimum value from the distance matrices of reference genomes and bins is taken as the representative of the distance of the gene *g*, respectively. For genes that cannot be aligned in ref genomes or bins, the distance value between the gene and the corresponding group is assigned as “1,” indicating a distant relationship:

$$d_{\mathrm{rep}}(g)=\min\left(D_{\mathrm{phylogenetics}}^{\mathrm{ref\_genomes}}(g),D_{\mathrm{phylogenetics}}^{\mathrm{bins}}\left(g\right)\right),$$

*d*(*g*) = 1 (if gene *g* is unaligned).

Similarly, the set of distance values for all genes in the target BGC, with respect to the reference genomes and bins, was obtained:

$$D_{\mathrm{target\_BGC}}=\{d_{\mathrm{rep}}\left(g_{1}\right),d_{\mathrm{rep}}\left(g_{2}\right),d_{\mathrm{rep}}\left(g_{3}\right),\ldots ,d_{\mathrm{rep}}(g_{n})\}.$$

Finally, the distance between the two groups (reference genomes and bins) was statistically tested by paired *t*‐test or Wilcoxon test to determine any significant differences.

### Construction of simulated HTBGCs and metagenomes

Simulated HTBGCs and metagenomes were constructed according to the following steps. First, 20 reference genomes of keystone species were selected from previous research [34, 35]. Second, the BGC composition of each genome was identified using antiSMASH (V6.1.1) [56]. Third, 10 reference genomes were selected as the data set for each simulation, and 10 BGCs identified from the reference genomes were randomly selected and inserted into other genomes with 2% mutations. This procedure was repeated ten times at each library size. Fourth, the resulting genomes after insertion were converted into paired‐end metagenomic reads using InSilicoSeq [60]. InSilicoSeq generates reads from multiple genomes according to a default log‐normal abundance distribution with other distributions built‐in, to provide exact abundances for each genome. By various sequencing library sizes, a total of 30 simulated metagenomes [(10 unique sets) × (3 library sizes: 50 million, 100 million, and 200 million paired‐end reads)] were constructed.

### Prediction of HTBGCs in simulated metagenomes

First, MAGs were constructed from each simulated metagenome using metaWRAP pipeline (V1.2.1) [54] with default parameters. HTBGCs were subsequently detected in the resulting MAGs using HTBGC‐Finder. The detection results were then compared to the simulated insertion results to evaluate the prediction performance of HTBGC‐Finder.

The F1 score, used for evaluating HTBGC‐Finder prediction accuracy, was calculated based on HTBGCs successfully identified by antiSMASH, an integral component of the HTBGC‐Finder tool. The F1 score was defined as the harmonic mean of precision and recall, and was calculated as follows:

$$F_{1}=\frac{2}{\frac{1}{\mathrm{precision}}+\frac{1}{\mathrm{recall}}},$$

where

$$\mathrm{precision}=\frac{\mathrm{TP}}{\mathrm{TP}+\mathrm{FP}}\;\text{and}\;\mathrm{recall}=\frac{\mathrm{TP}}{\mathrm{TP}+\mathrm{FN}}.$$

Here, for a given metagenome, TP (true positives) is the number of BGCs that were correctly predicted to be horizontally transferred; FP (false positives) is the number of BGCs that were incorrectly predicted to be horizontally transferred; and FN (false negatives) is the number of BGCs that were missed, that is, incorrectly predicted as not being horizontally transferred by HTBGC‐Finder.

### Figures and statistical analysis

RStudio (V2023.12.1) running R (V4.3.3) [61] was employed for generating figures. Statistical significance for normally distributed variables was estimated by student's *t‐* tests, and non‐normally distributed variables were analyzed by the Wilcoxon rank‐sum test. Chi‐squared test and Fisher's exact test were used to analyze contingency tables depending on specific grouping conditions. All comparisons were two‐sided with an alpha level of 0.05.

The GCF network was visualized by networkD3 (V0.4) [62], htmlwidgets (V1.6.4) [63], and webshot (V0.5.5) [64]. The evolutionary tree was visualized using Evolview (V3) [65]. The ROC curve was drawn by pROC (V1.18.5) [66]. The box plot, bar chart, and layer pie plot were drawn by ggplot2 (V3.4.2) [67]. The alluvial figures were drawn by ggalluvial (V0.12.5) [68]. The rate of potential horizontal transfer among different groups was compared using the Chi‐squared test by ggstatsplot (V0.12.1) [69] or Fisher's exact test, and the figures were performed by ggplot2 (V3.4.2) [66] and ggbreak (V0.1.2) [70]. The chord diagram is plotted with circlize [71] (V0.4.16). The software packages used in this study are free and open source.

## AUTHOR CONTRIBUTIONS

**Lei Zhang**: Conceptualization. **Jia‐cheng Wu** and **Xiao Yang**: Methodology; software. **Xiao Yang**, **Jia‐cheng Wu**, and **Lan‐lan Zhao**: Data curation. **Xiao Yang** and **Jia‐cheng Wu**: Writing—original Draft. **Lei Zhang**, **Zi‐yun Li**, and **Guo‐ping Zhao**: Writing—review & editing. All authors have read the final manuscript and approved it for publication.

## CONFLICT OF INTEREST STATEMENT

The authors declare no conflicts of interest.

## ETHICS STATEMENT

No animals or humans were involved in this study.

## Supporting information

The online version contains supplementary figures and tables available.

Figure S1 Average nucleotide identity result of BGCs from reference genomes and bins clustered with thiopeptide into the same gene cluster family.

**Table S1:** BioSample associated with the previously identified thiopeptides.
**Table S2:** Detailed analysis of potential horizontally transferred BGCs of a subset dataset of Human Microbiome Project (HMP).
**Table S3:** Reference genomes corresponding to the taxonomy profiling of the MAG containing horizontally transferred thiopeptide.
**Table S4:** Average nucleotide identity result of the selected HMP genomes and MAGs.
**Table S5:** Detection results of simulated metagenomes using HTBGC‐Finder.
**Table S6:** F1 scores of BGC prediction in simulated metagenomes.
**Table S7:** Samples retrieved from the human gut and the corresponding MAGs.
**Table S8:** Detailed information for potential horizontally transferred BGCs of human gut microbiota.
**Table S9:** Detailed information for all BGCs identified from MAGs of human gut microbiota.

## Data Availability

The data that supports the findings of this study are available in the supplementary material of this article. HTBGC‐Finder is an open‐source command‐line tool that is implemented in Python and Bash. The source code and complete instructions for installation and usage are freely available online at https://github.com/Shirly-Yang/HTBGC-Finder. The data and scripts used are saved in GitHub https://github.com/Shirly-Yang/2025iMetaOmics. Supplementary materials (figures, tables, graphical abstract, slides, videos, Chinese translated version, and update materials) may be found in the online DOI or *iMetaOmics* Science http://www.imeta.science/imetaomics/.
