# Supplementary material for: Systematic characterization of horizontally transferred biosynthetic gene clusters in the human gut microbiota using HTBGC‐Finder
Source: IMetaOmics. 2025 Feb 1;2(1):e62. doi: 10.1002/imo2.62 (PMC12806405; doi:10.1002/imo2.62)
Supplement: Supplementary file 1 — Figure S1 Average nucleotide identity result of BGCs from reference genomes and bins clustered with thiopeptide into the same gene cluster family. [file IMO2-2-e62-s002.docx]

**Supporting information to**

**Systematic Characterization of Horizontally Transferred Biosynthetic Gene Clusters in the Human Gut Microbiota Using HTBGC-Finder**

**Running title:** Characterizing Horizontally Transferred BGCs in Human Gut Using HTBGC-Finder

Jiacheng Wu^a,c,#^, Xiao Yang^a,#^, Lanlan Zhao^a^, Ziyun Li^a,b^, Guoping Zhao^a,b,c,*^, Lei Zhang^a,b,*^

^a^Microbiome-X, School of Public Health, Cheeloo College of Medicine, Shandong University, Jinan, 250000, China

^b^State Key Laboratory of Microbial Technology, Shandong University, Qingdao, 266000, China

^c^CAS Key Laboratory of Computational Biology, Bio-Med Big Data Center, Shanghai Institute of Nutrition and Health, University of Chinese Academy of Sciences, Shanghai, 200000, China

^#^ These authors contributed equally: Jia-Cheng Wu, Xiao Yang

*Correspondence: [gpzhao@sibs.ac.cn](mailto:gpzhao@sibs.ac.cn) (Guo-Ping Zhao), [zhanglei7@sdu.edu.cn](mailto:zhanglei7@sdu.edu.cn) (Lei Zhang)

**Figure S1 Average nucleotide identity(ANI) result of reference genomes and bins clustered with thiopeptide into the same gene cluster family.** The reference genomes are represented by the strain names and GenBank genome accessions, and metagenomic data is represented by metagenome-assembled genomes (MAGs) accession number. MAG “SRS048870 bin18” contains the target thiopeptide.
